# In Vitro Antioxidant Properties, Glucose-Diffusion Effects, α-Amylase Inhibitory Activity, and Antidiabetogenic Effects of *C. Europaea* Extracts in Experimental Animals

**DOI:** 10.3390/antiox10111747

**Published:** 2021-10-31

**Authors:** Hayat Ouassou, Mohamed Bouhrim, Noureddine Bencheikh, Mohamed Addi, Christophe Hano, Hassane Mekhfi, Abderrahim Ziyyat, Abdekhaleq Legssyer, Mohamed Aziz, Mohamed Bnouham

**Affiliations:** 1 Laboratory of Bioresources, Biotechnology, Ethnopharmacology and Health, Faculty of Sciences, Mohammed First University, B.P. 717, Oujda 60040, Morocco or h.ouassou@ump.ac.ma (H.O.); or m.bouhrim@ump.ac.ma (M.B.); or b.noureddine@ump.ac.ma (N.B.); or h.mekhfi@ump.ac.ma (H.M.); or a.ziyyat@ump.ac.ma (A.Z.); or a.legssyer@ump.ac.ma (A.L.); or m.aziz@ump.ac.ma (M.A.); or m.bnouham@ump.ac.ma (M.B.); 2 Laboratoire d′Amélioration des Productions Agricoles, Biotechnologie et Environnement, (LAPABE), Faculté des Sciences, Université Mohammed Premier, Oujda 60000, Morocco; 3 Laboratoire de Biologie des Ligneux et des Grandes Cultures, INRAE USC1328, Eure et Loir Campus, Orleans University, 28000 Chartres, France; christophe.hano@univ-orleans.fr

**Keywords:** *Caralluma europaea*, antidiabetogenic effect, hyperglycemia, glucose diffusion, α-amylase, antioxidant

## Abstract

*Caralluma europaea* (Guss.) N.E.Br. (*C. europaea*), is a medicinal plant used traditionally to treat diabetes mellitus (DM) in Morocco. This study aimed to investigate the in vitro antioxidant properties, glucose diffusion effects, α-amylase inhibitory activity, and pancreatic protective effects of *C. europaea* in experimental alloxan-induced diabetes in mice. Total phenolic contents were determined by Folin–Ciocalteu colorimetric method, total flavonoid contents were measured by aluminum chloride colorimetric assay, and tannins contents were determined by employing the vanillin method. *C. europaea* ethyl acetate fraction exhibited high antioxidant potential in terms of radical scavenging (DPPH) (IC50 = 0.22 ± 0.01 mg/mL), β-carotene bleaching activity (IC50 = 1.153 ± 0.07 mg/mL), and Ferric-reducing antioxidant power. Glucose diffusion was significantly inhibited by the ethyl acetate fraction at 60,120and 180 min, while the aqueous extract did not have this inhibitory effect when compared with the control group. Potent α-amylase inhibitory activity was observed in the ethyl acetate fraction and the aqueous extract in vitro and in vivo using STZ-diabetic rats. On the other hand, the administration of the ethyl acetate fraction (60 mg/kg) significantly attenuated alloxan-induced death and hyperglycemia in treated mice. Furthermore, histopathological investigations revealed that the ethyl acetate fraction protected islets of Langerhans against alloxan-induced tissue alterations. These results suggest that *C. europaea* exhibited an important antihyperglycemic effect via the inhibition of glucose diffusion and pancreatic α-amylase activity. In addition, the antidiabetogenic effect of *C. europaea* might be attributed to their polyphenol and flavonoid compounds, which could be reacted alone, or in synergy, to scavenge the free radicals produced by the alloxan.

## 1. Introduction

Diabetes mellitus (DM), is a group of metabolic disorders that is characterized by a high blood glucose level (hyperglycemia), mainly caused by insufficient insulin production or unresponsiveness of the body to insulin or both [1,2]. The postprandial hyperglycemia takes part in the development of numerous diabetes complications such as nephropathy, retinopathy, neuropathy, and diabetic foot ulcer [3,4]. The control of postprandial hyperglycemia is one of the vital alternatives to the management of chronic hyperglycemia in diabetic patients [5,6]. This is accomplished by inhibiting the enzymes involved in the digestion of carbohydrates such as α-amylase [6,7]. Therefore, inhibiting α-amylase could be an effective way to reduce starch digestibility, decrease the rate of glucose absorption, and consequently reduce postprandial hyperglycemia [8,9,10]. In addition to this, it has been well documented that oxidative stress plays a major role in the pathology of diabetes complications of both types of diabetes mellitus [11,12]. Oxidative stress is an imbalance between the production of oxygen reactive species (ROS) and antioxidant defenses [13]. It results from high concentrations of reactive oxygen species (ROS) and/or reactive nitrogen species (RNS) [9]. It is believed that oxygen reactive species have been implicated in the etiology and development of vascular complications in diabetes [14,15,16]. Free radical formation in diabetes leads to damage of the biomolecules such as lipids, proteins, and nucleic acids, and induces the appearance of long-term diabetic complications [17,18]. There are various approaches to treat and prevent diabetes and its vascular complications; one of them is herbal remedies. Therefore, there is an increasing interest in evaluating herbal medicines, which are seen to be safer and to have negligible side effects [19]. The most popular natural antioxidants are vitamin C, vitamin E, flavonoids, carotenoids, and phenolic compounds [20]. Antioxidants, and vitamins such as vitamin C and E, are effective in reducing oxidative stress in experimental diabetes [21,22]. Moreover, vitamin C has also been shown to reduce glycosylated hemoglobin in the diabetic patient [23]. Over the past decade, evidence has accumulated that plant products have been shown to have an important antioxidant activity [24,25]. *Caralluma europaea* (Apocynaceae family) is a leafless, succulent and angular plant distributed in Morocco, Egypt, Spain, Italy, Libya, Tunisia, and Algeria [26,27]. In Moroccan traditional medicine, it is used for antidiabetic properties [28,29]. Moreover, various extracts of aerial parts *C. europaea* showed antidiabetic activities in an animal model for T1DM [30,31]. Besides, *C. europaea* was also found to be more effective in inhibiting the activities of α-glucosidase and α-amylase (key target enzymes for T2DM) [30,32]. Hence, the current study was undertaken to appraise the in vitro antioxidant and antihyperglycemic potential of *C. europaea* through α-amylase inhibitory activities and glucose diffusion. We also attempted to probe, for the first time, the potential protective effect of *C. europaea* against chemically induced diabetes mellitus in mice.

## 2. Materials and Methods

### 2.1. Chemicals and Reagents

The following drugs and solvents were used in this study: 2, 2-diphenyl-1-picrylhydrazyl [DPPH] (Alfa Aesar, Hamburg, Germany), L-ascorbic acid (Sigma Aldrich, Dorset, UK), linoleic acid (Sigma Aldrich, St. Louis, MO, USA), Tween 40 (Sigma Aldrich, St. Louis, MO, USA), β-carotene (Sigma Aldrich, St. Louis, MO, USA), Potassium ferricyanide K3[Fe(CN)6] (Sigma Aldrich, St. Louis, MO, USA),Trichloroacetic acid (TCA) (Sigma Aldrich, St. Louis, MO, USA), Ferric chloride FeCl3 (Sigma Aldrich, St. Louis, MO, USA), Alloxan monohydrate (Allx monohydrate 98%, ACROS Organics), Streptozotocin (Sigma-Aldrich, Hamburg, Germany), α-amylase and acarbose (Sigma Aldrich, St. Louis, MO, USA), glucose oxidase–peroxidase (GOD–POD) (Biosystems, Barcelona, Spain), starch (Sigma Aldrich, Riedel-de Haen, Germany), Folin–Ciocalteu, gallic acid, aluminum chloride, and quercetin (Sigma Aldrich, St. Louis, MO, USA). The solvents utilized were chloroform (Sigma Aldrich, Riedel-de Haen, Germany), diethyl ether (Sigma Aldrich, Riedel-de Haen, Germany), and methanol (Sigma Aldrich, Riedel-de Haen, Germany). All chemicals and solvents used were analytical grades.

### 2.2. Plant Material and Extraction Method

The aerial part of *C. europaea* was collected from Oujda city, Morocco, in May 2017. The plant was identified by the Botanist Pr. Mohamed Fennane with a voucher specimen numbered HUMPOM 150, and kept in the herbarium of the Faculty of Sciences, University Mohamed First, Oujda, Morocco.

#### 2.2.1. Preparation of the *C. europaea* Aqueous Extract

The plant material was air-dried, cut into small pieces and then ground into a fine powder. Biomass (200 g) was extracted by maceration with 600 mL of distilled water for 24 h and filteration through filter paper. Then, the mixture was evaporated at 45 °C using a rotary evaporator under a vacuum. The extract was preserved at −20 °C until use.

#### 2.2.2. Fractionation

A quantity of 100 g of dried, powdered *C. europaea* underwent sequential and successive extractions in a Soxhlet apparatus by using different solvents (700 mL) of increasing polarity. Each solvent fraction was evaporated at 45 °C using a rotary evaporator under vacuum and pressurized conditions until dry. The solvents used were, in order, hexane, dichloromethane, ethyl acetate, methanol and distilled water. The yields of these four organic fractions were 11.3%, 5.4%, 2.6%, 22.1%, and 5.9%, respectively. The ethyl acetate fraction of *C. europaea* (EACe) was determined to be active and was used in the rest of the experiments.

### 2.3. Experimental Animals

The current study was performed on normal adult Wistar rats and Swiss albino mice from the local animal husbandry of the faculty of Science, Mohammed First University, Oujda, Morocco. All animals were housed in cages with standard laboratory conditions (12/12 h light/dark cycle at 23 ± 2 °C). They were provided with feed and tap water ad libitum. All animal experiments were carried out following the Guide for the Care and Use of Laboratory Animals, published by the US National Institutes of Health (NIH Publication No. 85-23, Revised in 1985).

### 2.4. Total Phenolic Content

The total phenolic content of the ethyl acetate fraction of *C. europaea* (EACe) was determined by the Folin–Ciocalteu method with slight modifications [33]. The concentration gradient of gallic acid was prepared as a standard solution (0; 20; 40; 60; 80 and 100 µg/mL), and a calibration curve was determined using gallic acid. The test sample (200 µL of EACe) or gallic acid was added to 1000 µL Folin–Ciocalteu reagent and mixed for 5 min, followed by the addition of 800 µL of sodium carbonate solution (75 g/L). The mixture was incubated in the dark for 60 min. All samples were performed in triplicate. The absorbance of the mixture was measured at 765 nm against methanol as blank. The results were expressed as mg of gallic acid equivalents (GAE) per gram of dry matter of sample.

### 2.5. Total Flavonoid Content

The content of total flavonoids of the EACe extract was measured by the aluminum chloride colorimetric method with some modifications [34]. Quercetin standard solution was prepared with methanol (0; 20; 40; 60; 80 and 100 µg/mL). In brief, 200 µL of EACe fraction or quercetin was added to 1000 µL of distilled water. Then, 50 µL of 5% NaNO_2_ was added. After 6 min, 120 µL of 10% AlCl_3_ was added to the mixture. Immediately, the mixture was incubated for 5 min, followed by the addition of 400 µL of NaOH (1 M), and 230 µL of distilled water. The absorbance was determined at 510 nm using methanol as a blank. All trials were performed in triplicate. The total flavonoid content of the extract was expressed as mg of quercetin equivalents (QE) per gram of dry matter of sample. 

### 2.6. Total Tannins Content

Condensed tannins were evaluated according to the method described by Sun et al. [35] with slight modifications. Briefly, 500 µL of EACe was added to 3 mL of 4% vanillin (*w*/*v*) in methanol and the tubes were carefully agitated with a mixer. Then, a volume of 1.5 mL of concentrated HCl was added and the tubes were agitated again. The mixture was allowed to stand for 15 min, and the absorption was measured at 500 nm against methanol as a blank. The results were plotted after the gallic acid standard was made in the same manner (0, 0.125, 0.25, 0.5 and 1 mg/mL). The total tannincontent was expressed as mg of gallic acid equivalents (GAE) per gram of dry matter of sample. All samples were performed in triplicate.

### 2.7. Antioxidant Assays

#### 2.7.1. DPPH Radical-Scavenging Activity

The free-radical scavenging activity of *C. europaea* was measured by using a 1,1-diphenyl-2-picryl-hydrazyl (DPPH) assay, as described by Chu et al. with some modifications [36]. Briefly, l mL of increasing concentrations of EACe fraction (0.25; 0.5; 1.25 and 2.5 mg/mL) was added to l mL of 0.1 mM DPPH radical solution. The reaction mixture was incubated in the dark at room temperature for 30 min. The absorbance was then measured at 517 nm.Ascorbic acid was used as a positive control. Redundant testing was performed three times with each sample (*n* =3). The scavenging activity of the samples was calculated according to the following formula:

SA = (1 − Abs in the presence of sample/Abs in the absence of sample) × 100

#### 2.7.2. β-Carotene/Linoleic Acid β-Bleaching Assay

The determination of antioxidant activity was evaluated according to Sun et al. [37]. A solution of β-carotene (0.1 mg/mL) in chloroform (1 mL), (20 mg) linoleic acid, and (200 mg) Tween 40 were transferred into a round-bottom flask. After evaporation (40 °C) of chloroform in a rotary evaporator, (50 mL) distilled water was added and the resulting mixture was vigorously stirred for 30 min. A total of 200 μL of EACe fraction at increasingconcentrations (0.5; 1; 2.5 and 5 mg/mL) was added to the mixture (2.5 mL) in the separated test tubes. Then, the tubes were incubated in a hot water bath at 50 °C. After 2 h. the absorbance was measured at 490 nm. All samples were performed in triplicate. Ascorbic acid was used as a positive control.

The antioxidant activity (AA) was presented as a percentage of inhibition relative to the control using the following equation:

AA = [(DR control − DR sample)/DR control] × 100

#### 2.7.3. Ferric-Reducing Antioxidant Power (FRAP) Assay

The reducing power of the EACe fraction was determined according to the method described by Karagozler et al. [38]. In brief, 1 mL of EACe fraction at different concentrations (0.1; 0.2; 0.4; 0.6; 0.8 and 1 mg/mL) was mixed with 2.5 mL of phosphate-buffered solution (0.2 M, pH = 6.6) and 2.5 mL of 1% of potassium ferrocyanide K_3_Fe(CN)_6_. The mixture was incubated at 50 °C for 30 min. After the incubation, 2.5 mL of 10% trichloroacetic acid (TCA) was added, and the mixture was centrifuged at 3000 rpm for 10 min. Finally, 2.5 mL of supernatant was mixed with 2.5 mL of distilled water and 0.5 mL of FeCl_3_6H_2_O (0.1%). The absorbance was recorded at 700 nm. Higher value absorbance of the reaction mixture indicated increased reducing power.

### 2.8. Effect of C. europaea Extracts on in Vitro Glucose Movement

In vitro glucose diffusion was measured using a slightly modified version of the method of Gallagher et al. [39]. In brief, the model consisted of a one-sided sealed dialysis tube (SpectraPore^®^, 2000 Da, 6 cm × 11.5mm) into which 3 mL of glucose solution (22 mmol L^−1^) in NaCl (0.15 mol L^−1^) was mixed with 1 mL (5 mg/kg) of each of the following *C. europaea* extracts: AECe, HCe, DCe, EACe, MCe, and ACe. The control group (without extract, with distilled water) was also used for comparison. The dialysis membrane of each sample was sealed at each end and was placed in a 50 mL centrifuge tube containing 45 mL of NaCl solution (0.15 mol L^−1^). The tubes were placed in a shaking water bath at 37 °C. The glucose content in the external medium was determined after 30, 60, 120, and 180 min using a glucose oxidase–peroxidase assay kit. Distilled water was used as the control. 

### 2.9. Inhibition of α-Amylase Activity In Vitro

The pancreatic α-amylase inhibition assay was investigated according to the method described by Daoudi et al. [7], with slight modifications. Briefly, 200 µL of 0.02 M phosphate buffer (pH = 6.9); 200 µL of α-amylase enzyme solution (13 IU); and 200 µL of *C. europaea* extracts (0.05, 0.1, 0.2, and 0.3 mg/mL) or acarbose (0.018 and 0.023 mg/mL, positive control) were preincubated at 37 °C for 10 min. Then, 200 µL of starch (1%) solution (dissolved in the above buffer) was added to test tubes and the mixture was incubated at 37 °C for 20 min. The reaction was terminated by adding 600 µL of dinitrosalicylic acid (DNSA) color reagent (2.5%) followed by incubation in a hot water bath at 100 °C for 8 min to inactivate the enzymes. Afterward, the tubes were put in a cold water bath, after which 1 mL of distilled water was added. The absorbance was measured at 540 nm. The α-amylase inhibitory activity (%) was calculated using the formula below:

% Inhibition = ((DO Control (540 nm) − DO Sample (540 nm))/DO Control (540 nm)) × 100

### 2.10. Inhibition of α-Amylase Activity In Vivo

#### 2.10.1. Diabetes Induction

After overnight fasting, diabetes was induced by intraperitoneal injection of streptozotocin (STZ) freshly dissolved in citrate buffer (0.1 m citrate and 0.1 m phosphate, pH 4.5) at a dose of 60 mg/kg. The animals were allowed to drink glucose solution overnight to avoid hypoglycemia and death. One week after STZ administration, the blood glucose was measured and the animals with fasting blood d-glucose greater than or equal to 1.5 g/L were considered to have diabetes 

#### 2.10.2. Experimental Design

Fasted rats were divided into four groups consisting of six rats each (*n* = 6). The control groups received only distilled water orally at a dose of 10 mL/kg, the AeCe groups received extract at a dose of 250 mg/kg, the EACe groups received extract at a dose of 50 mg/kg, and the acarbose groups received acarbose at a dose of 10 mg/kg. All rats were orally loaded with starch (2 g/kg) at 30 min after treatments. Blood samples were collected from the venous section of the tail from rats under light ether anesthesia at 0, 30, 60, and 120 min to measure glycemia.

### 2.11. Effect of Ethyl Acetate Fraction of C. europaea on Allx-Induced Diabetic Mice

#### 2.11.1. Diabetes Induction

Diabetes was experimentally induced by a single intraperitoneal injection of Alloxan monohydrate at 100 mg/kg (body weight) in mice. Alloxan was freshly prepared in phosphate–citrate buffer (pH = 4.5) and injected into overnight fasted mice. One week after the injection, the blood glucose was measured and the animals were considered to have diabetes if the fasting blood glucose value was greater than or equal to 1.5 g/L. 

#### 2.11.2. Experimental Design

The animals were divided into five groups: control group, Allx group, Allx + EACe (60 mg/kg), and Allx  +  Asco (40 mg/kg) group, respectively. Overthree days, treated mice orally received EACe fraction (60 mg/kg) and ascorbic acid (40 mg/kg) according to their respective groups, followed by a single intraperitoneal injection of alloxan monohydrate at a 1h time interval. Control and Allx groups received oral administration of distilled water (10mL/kg of body weight) followed by an intraperitoneal injection of phosphate–citrate buffer and Alloxan, respectively. The treatment was sustained for a week after Alloxan administration. The feeding experiment was carried out for a week (7 days) after the induction of diabetes. Fasting blood glucose and body weight were recorded on the beginning and the termination day of the treatment. 

#### 2.11.3. Histological Analysis of Pancreatic Tissues

The pancreases of mice were prepared for the examination of the microscopic lesions. All pancreatic tissues were preserved in 10% buffered formalin solution (48 h), embedded in paraffin, and then cut into 4 to 5 μm sections using a rotating microtome (microtome leitz 1512). Pancreas sections were stained with hematoxylin and eosin standard method. Stained sections of the pancreas were qualitatively (morphological) analyzed and visualized under optical microscopy (Optika Microscopes, Italy), and captured by an Infinity camera microscope with ×40 magnification.

### 2.12. Statistical Analysis

Data were expressed as means ± standard errors. The statistical analysis was performed using GraphPad Prism 5 (GraphPad Software), San Diego, CA, USA. One-way ANOVA was used to determine the significant differences between the means of multiple groups. The differences between the means were considered significant at the probability level *p* < 0.05. 

## 3. Results

### 3.1. Total Phenolic, Flavonoid, and Tannin Contents of C. europaea

The total phenolic and flavonoid contents of EACe are presented in Table 1. The results were determined from calibration curves of gallic acid (*Y* = 0.0123*x* + 0.0004, R^2^ = 0.993), quercetin (*Y* = 0.1638*x* + 0.0193, R^2^ = 0.9839), andgallic acid (*Y* = 0.1185*x*, R^2^ = 0.9819), respectively. The content of phenolic compounds in ethyl acetate fraction was 7.159 ± 0.35 mg GAE/g, while the total flavonoid content was 1.523 ± 0.01 mg QE/g. A lower quantity of tannin content was observed in EACe, with 0.097 ± 0.002 mg GAE/g Extract. 

### 3.2. Antioxidant Activity

#### 3.2.1. DPPH-Radical Scavenging Activity

**Figure 1:**
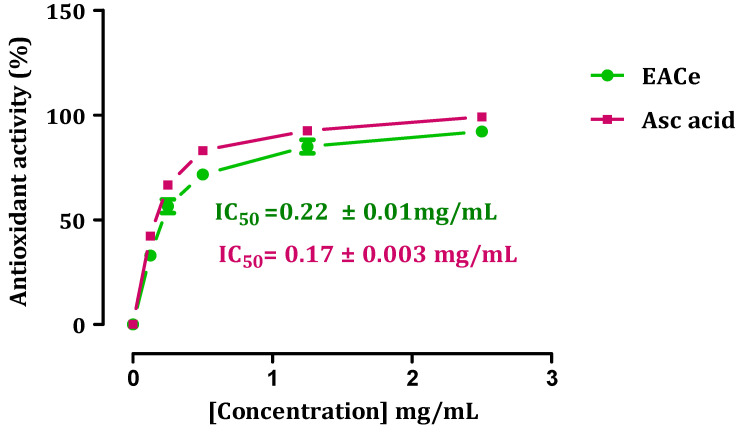
DPPH-scavenging activity of ethyl acetate fraction of *C. europaea* (EACe). Each value is expressed as mean ± SEM (*n* = 3).

The antioxidant capacity of the ethyl acetate fractions from *C. europaea* was determined by measuring their ability to scavenge DPPH radicals (Figure 1). The results were expressed as IC_50_ (mg/mL), which is the amount of antioxidant necessary to decrease the initial DPPH radical by 50% [40]. A lower IC_50_ value corresponds with a higher antioxidant power. An excellent antioxidant activity was found in the ethyl acetate fraction, with an IC_50_ = 0.22 ± 0.01 mg/mL. By comparison, the standard ascorbic acid had free-radical scavenging activity of IC_50_ = 0.17 ± 0.003 mg/mL. In this study, the reduction of DPPH occurred in a concentration-dependent manner (dose–response), as observed from the high reduction of DPPH (higher radical activity) at 2.5 mg/mL concentrations. The scavenging activity increased significantly with increasing EACe concentrations up to a certain dose, 2.5 mg/mL, and then leveled off with a maximum of 98% of the antioxidant effect. 

#### 3.2.2. β-Carotene Bleaching Method

**Figure 2:**
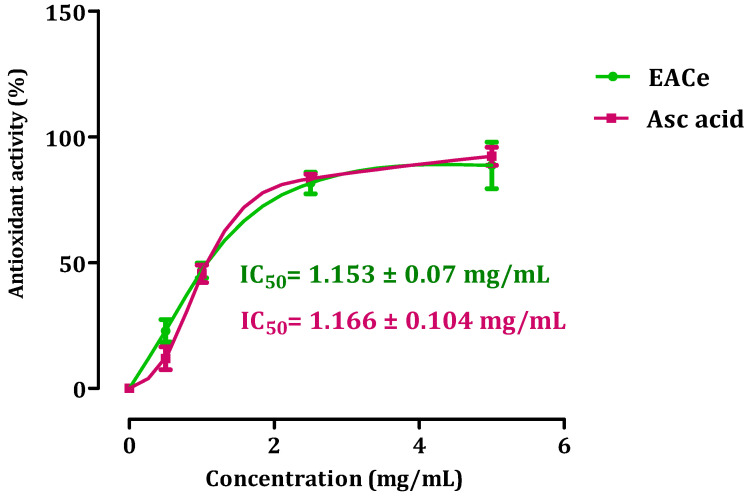
Antioxidant activity of ethyl acetate fraction from *C. europaea* (EACe). Each value is expressed as mean ± SEM (*n* = 3).

The β-carotene bleaching technique is based on the loss of the orange color due to its reaction with radicals formed by linoleic acid oxidation in an emulsion. Therefore, the rate of β-carotene bleaching can be slowed down in the presence of antioxidants [41]. The ability of EACe to inhibit the bleaching of β-carotene is presented in Figure 2. Ascorbic acid was used as the standard drug for the evaluation of antioxidant activity. The IC50 value of EACe indicated that *C. europaea* extract possessed stronger antioxidant potential (IC50 = 1.153 ± 0.07 mg/mL). A similar antioxidant activity was found in the ethyl acetate fraction (EACe) in comparison with the results obtained for ascorbic acid (IC50 = 1.166 ± 0.104 mg/mL).

#### 3.2.3. Reducing Power 

**Figure 3:**
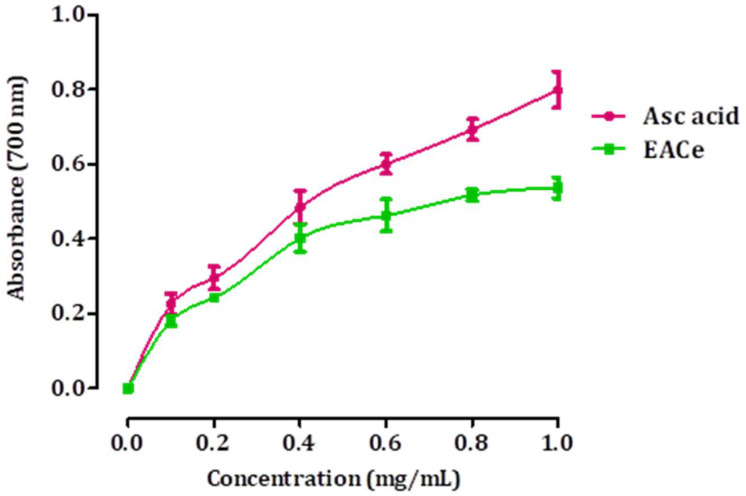
Reducing power of EACe at different concentrations. Each value represents a mean ± SD (*n* = 3).

The ferric-reducing antioxidant power method is based on the reduction of Fe^3+^ to Fe^2+^ ions in the presence of antioxidants in an acidic medium, which has an intense blue color and can be monitored by measuring the change in absorption at 593 nm. Increased absorbance is proportional to the antioxidant potency. The results obtained for the FRAP assay are presented in Figure 3. As shown, the ethyl acetate fraction from *C. europaea* (EACe) showed the highest reducing ability. It was found that the reducing power increased with concentrations of the EACe sample. Significantly, higher reducing power was observed at 1 g/mL. Ascorbic acid was utilized as a reference standard for the evaluation of the antioxidant activity. 

### 3.3. Effects of C. europaea on Glucose Diffusion In Vitro

**Figure 4:**
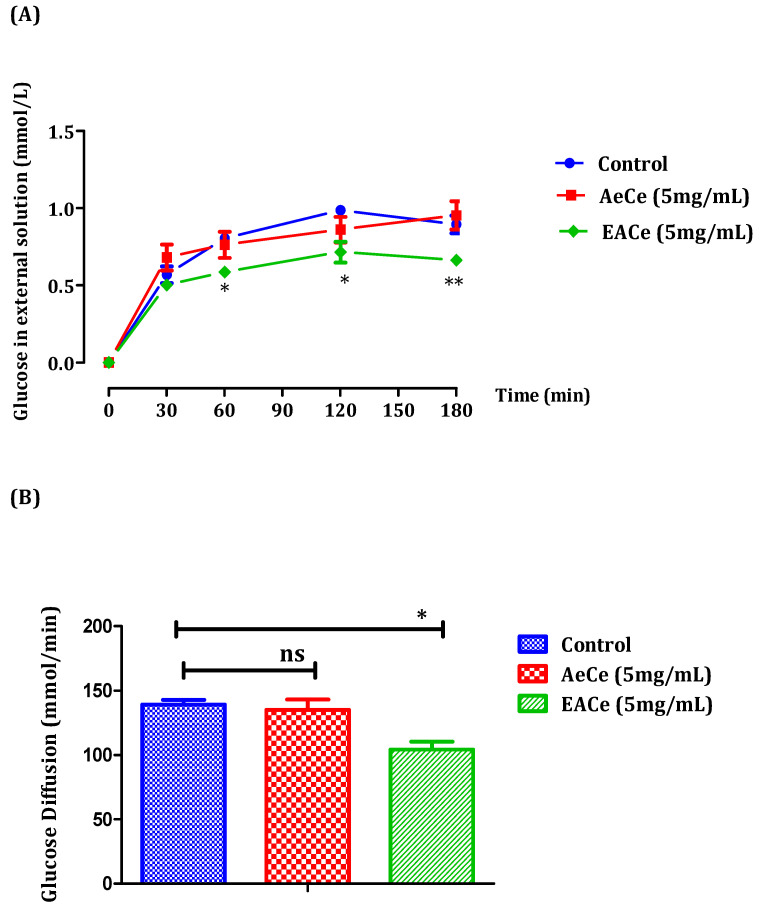
Effect of aqueous extract and fractions of *C. europaea* (5 mg/mL) on glucose diffusion through a dialysis membrane (**A**), and with a representation of the area under the curve of tested extracts (**B**). The values are the means ± SEM (*n* = 6). * *p* < 0.05, ** *p* < 0.01 compared to the control group.

In this study, the inhibitory effect on glucose movement in vitro was evaluated using a dialysis tube containing the extracts and glucose that had been soaked in NaCl solution. The diffusion of glucose into the external solution was measured by a glucose oxidase kit at 30, 60, 120, and 180 min. The effects of the aqueous extract (AECe) (5 mg/mL) and the ethyl acetate fraction (EACe) (5 mg/mL) from *C. europaea* on glucose diffusion are represented in Figure 3. The results obtained showed that the effect of AeCe was similar to the control. It did not significantly decrease glucose diffusion. It was evident from the graph that AeCe did not exhibit any significant inhibitory effect on glucose diffusion. However, the ethyl acetate fraction from *C. europaea* significantly inhibited glucose diffusion at 60, 120, and 180 min, and the glucose concentrations of the external solution were 0.58 ±0.02; 0.71 ± 0.06; and 0.66 ± 0.02 mmol/L, respectively, compared to the control group (Figure 4A). Additionally, the area under the curve for the AeCe group (135.03 ± 8.26 mmol/min) was not significantly different to the control group (139.30 ± 3.64 mmol/min), while the area under the curve for the EACe group was significantly lower (*p* < 0.05) (104.25 ± 6.18 mmol/min) than the area under the curve forthe control group (139.30 ± 3.64 mmol/min) (Figure 4B).

### 3.4. In Vitro Pancreatic α-Amylase Inhibition Assay

**Figure 5:**
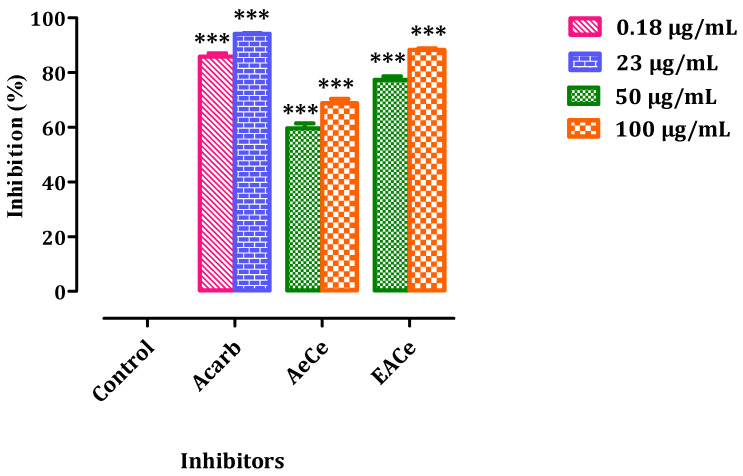
Inhibitory activity of the enzymes α-amylase by AeCe, EACe, and acarbose in vitro. The values are the means ± SEM (*n* = 3). AeCe: Aqueous extract of *C. europaea*; EACe: Ethyl acetate fraction of *C. europaea*. *** *p* < 0.001 compared to the control group.

The effects of AeCe and EACe on the activity of pancreatic α-amylase in vitro are represented in Figure 5. The α-amylase inhibitory potential of aqueous extract and ethyl acetate fraction from *C. europaea* was evaluated using starch as the substrate and acarbose as the positive control. Indeed, AeCe, EACe, and acarbose exhibited a dose-dependent enzyme inhibition. The ethyl acetate fraction exhibited a percentage inhibition of 77.30% and 88.29% at concentrations of 50 and 100 µg/mL, respectively. Likewise, the aqueous extract showed a percentage inhibition against α-amylase: 59.57% (50 µg/mL) and 68.79% (100 µg/mL). The standard drug acarbose displayed 85.81% and 94.14% inhibitions of α-amylase activity at concentrations of 0.18µg/mL and 32µg/mL, respectively. 

### 3.5. In Vivo Pancreatic α-Amylase Inhibition Assay

#### 3.5.1. Oral Starch Tolerance Test

##### Nondiabetic Rats

**Figure 6:**
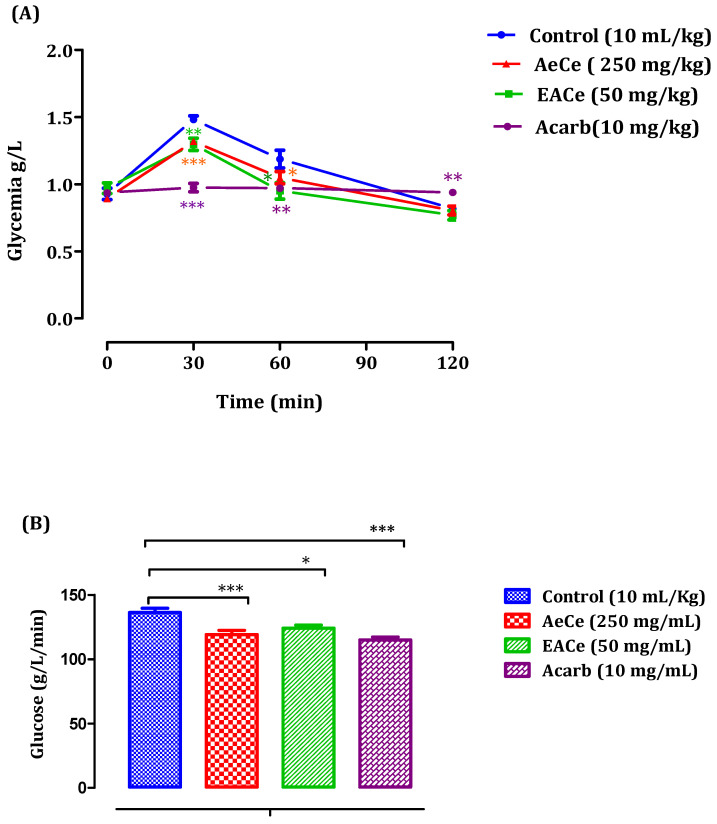
Effect of AeCe, EACe, and acarbose on plasma glucose levels after starch intake in normal rats (**A**), with a representation of the area under the curves (**B**). The values are the means ± SEM (*n* = 6). * *p* < 0.05, ** *p* < 0.01, and *** *p* < 0.001 compared to the control group.

Using normal rats, treated groups receiving 250 mg/kg of AeCe had significantly reduced postprandial hyperglycemia at 30 min (1.31 ± 0.02 g/mL) and 60 min (1.04 ± 0.04 g/mL). Likewise, the group treated with EACe with 50 mg/mL showed significant (*p* < 0.05, *p* < 0.01) decrease in blood glucose levels at 30 min (1.29 ± 0.04 g/L) and 60 min (0.95 ± 0.06 g/L), following starch loading. Therefore, no effect was observed at 120 min in all extract-treated groups when compared with the control group pretreated with distilled water (10 mL/kg). In addition, acarbose significantly (*p* < 0.01 and *p* < 0.001) reduced postprandial hyperglycemia during the two hours after starch loading at 30 min (0.97 ± 0.03 g/L), 60 min (0.97 ± 0.02 g/L), and 120 min (0.93 ± 0.02 g/L) when compared to the control group of rats pretreated with distilled water (Figure 6A). Additionally, the area under the curve (AUC d-glucose) was significantly (*p* < 0.05) lower in rats treated with AeCe (119.30 ± 3.07 g/L/min) and EACe (124.21 ± 2.33 g/L/min) than in rats treated with distilled water (136.35 ± 3.28 g/L/min). Additionally, the area under the curve of acarbose was significantly lower (115.08 ± 2.21 g/L/min) than the area under the curve of the water-treated rats (136.35 ± 3.28 g/L/min) (Figure 6B).

##### Diabetic Rats

**Figure 7:**
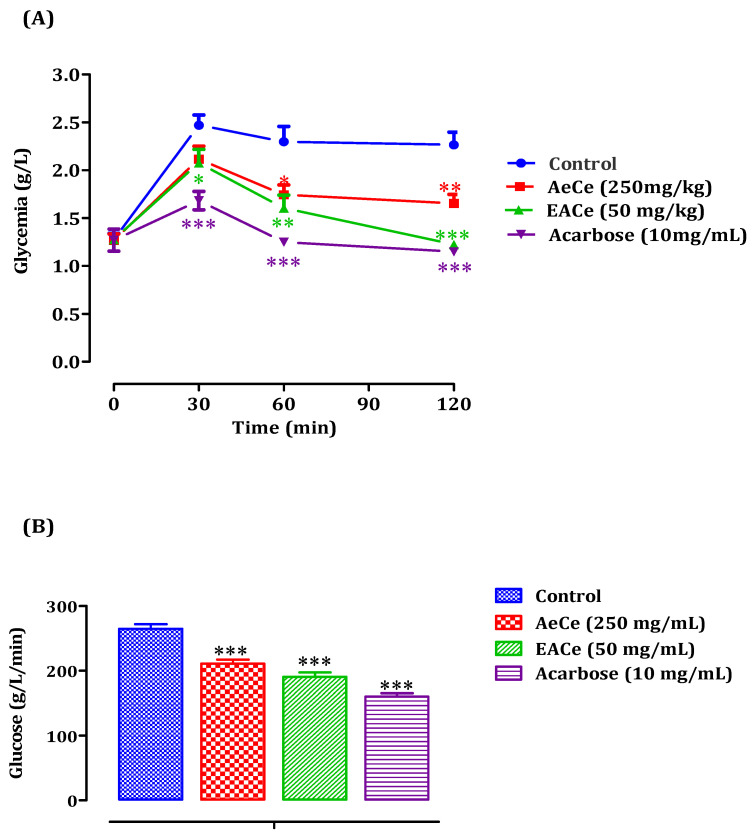
Effect of AeCe, EACe, and acarbose on plasma glucose levels after starch intake in diabetic rats (**A**), with a representation of the area under the curves (**B**). The values are the means ± SEM (*n* = 6). * *p* < 0.05, ** *p* < 0.01, and *** *p* < 0.001 compared to the control group.

The administration of AeCe at 250 mg/kg to STZ-induced diabetic rats significantly inhibited the blood glucose elevation after starch loading at 60 min (*p* < 0.05; 1.74 ± 0.10 g/L) and 120 min (*p* < 0.01; 1.65 ± 0.09 g/L) whencompared to the group of rats pretreated with distilled water in which the starch overloadinduced a remarkable peak in blood glucose at 30 min (2.47 ± 0.10 g/L), 60 min (2.29 ± 0.16 g/L), and 120 min (2.26 ± 0.13 g/L). Furthermore, EACe at 50 mg/kg significantly reducedpostprandial hyperglycemia at 30 min (*p* < 0.05; 2.07 ± 0.14 g/L), 60 min (*p* < 0.01; 1.60 ± 0.13 g/L), and 120 min (*p* < 0.001; 1.22 ± 0.009 g/L) whencompared to the control group. Similarly, acarbose at 10 mg/kg failed to suppress the peak blood glucose significantly (*p* < 0.001) at 30, 60, 120 min, and the glucose concentrations were 1.68 ± 0.09 g/L, 1.25 ± 0.02 g/L, and 1.14 ± 0.04 g/L, respectively (Figure 7A). Besides, the area under the curve (AUC d-glucose) was significantly (*p* < 0.001) lower in rats treated with AeCe (210. 55 ± 6.22 g/L/min) and EACe (190.35 ± 6.75 g/L/min) than in rats treated with distilled water (264.60 ± 6.87 g/L/min). Additionally, the curve area of acarbose was significantly (*p* < 0.001) lower (160.24 ± 4.82 g/L/min) than that of control rats (Figure 7B). 

### 3.6. Effect of Ethyl Acetate Fraction of C. europaea on Allx-Induced Diabetic Mice

#### 3.6.1. Effect of EACe on Body Weight and Survival Rate

**Figure 8:**
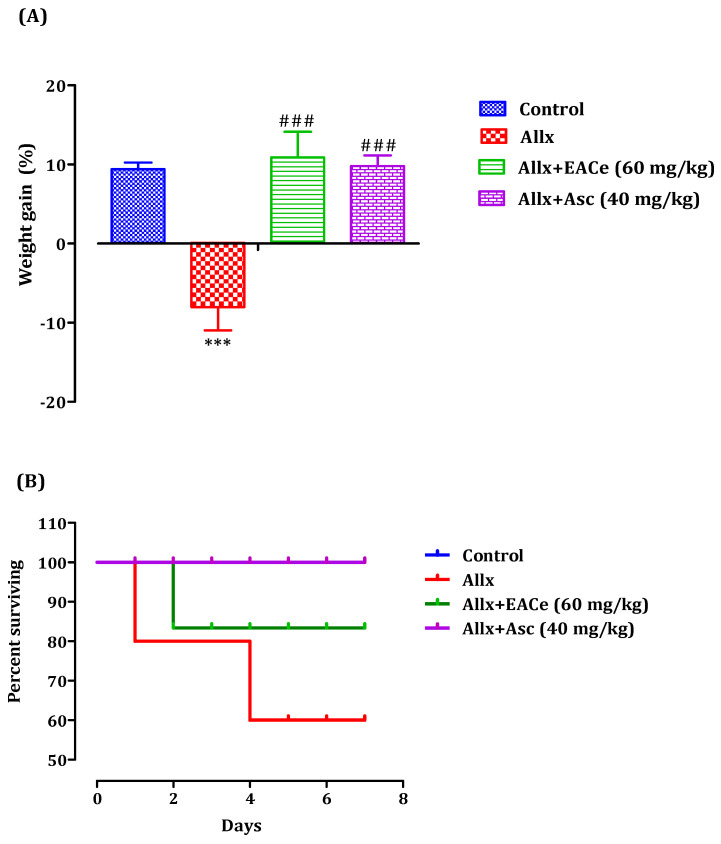
Effect of EACe on body weight (**A**), and survival rate of Swiss albino mice after alloxan administration (**B**). Data are means ± SEM, (*n* = 7). *** *p* < 0.001 compared to normal control group, ### *p* < 0.001 compared to alloxan group. EACe: Ethyl acetate fraction of *C. europaea*.

Body-weight loss has been associated with diabetes mellitus [42]. The changes in body weight in alloxan-induced diabetic mice were represented in Figure 8A. Alloxan-induced diabetic rats showed a significant decrease in body weight in the alloxan group (−12.01 ± 1.08%; *p* < 0.001) when compared to the normal control group (+8.94 ± 0.83%). However, the administration of EACe at 60 mg/Kg caused a significant increase in body weight (+12.43 ± 2.28%; *p* < 0.001) when compared to the alloxan group. Likewise, the pretreatment with ascorbic acid (40 mg/kg) group showed signs of an increase in body weight during the treatment period in comparison to the alloxan group. Inregards to survival rate, pretreatment with EACe upgraded the survival rate after alloxan injection (100 mg/kg) with a percentage of 85.72% compared to the Allx-group (57.14%) treated with Allx only. Additionally, the administration of ascorbic acid prevented mortality due to Allx administration when comparedto theAllx group (Figure 8B).

#### 3.6.2. Effect of EACe on Blood Glucose Level

**Figure 9:**
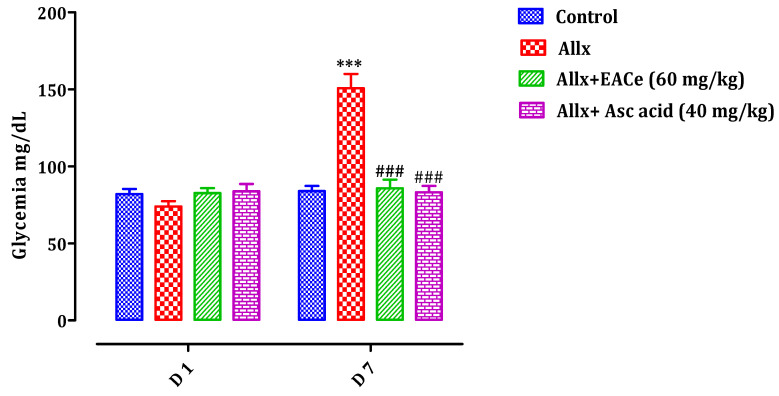
Effect of oral administration of EACe and ascorbic acid on blood glucose level before (D1) and after (D7) alloxan intraperitoneal injection in mice. Values represent the mean ± SEM, (*n* = 7). *** *p* < 0.001 compared to the normal control group at the corresponding time. ### *p* < 0.001 compared to alloxan group at the corresponding time. (D1 = Day 1; D7 = Day 7).

As shown in Figure 9, alloxan treatment induced a significant (*p* < 0.001) increase in fasting glucose level of the Allx group (150.75 ± 7.53 mg/dL) when compared with the normal control group (84.00 ± 3.13 mg/dL). However, pretreatment with EACe (60 mg/kg) with Allx in animals significantly reduced their blood glucose levels (85.5 ± 5.11 mg/dL; *p* < 0.001) when compared with those of the Allx group (150.75 ± 7.53 mg/dL). Additionally, treatment with ascorbic acid (40 mg/kg) induced a significant decrease in the fasting glucose level of theAsc-treated group (83.16 ± 4.53 mg/dL; *p* < 0.001) compared to the group treated with only alloxan. At the same time, no significant difference was found in blood glucose level between the EACe-treated group and Asc-treated group when compared to the normal control group.

#### 3.6.3. Histopathological Study of Pancreas

**Figure 10:**
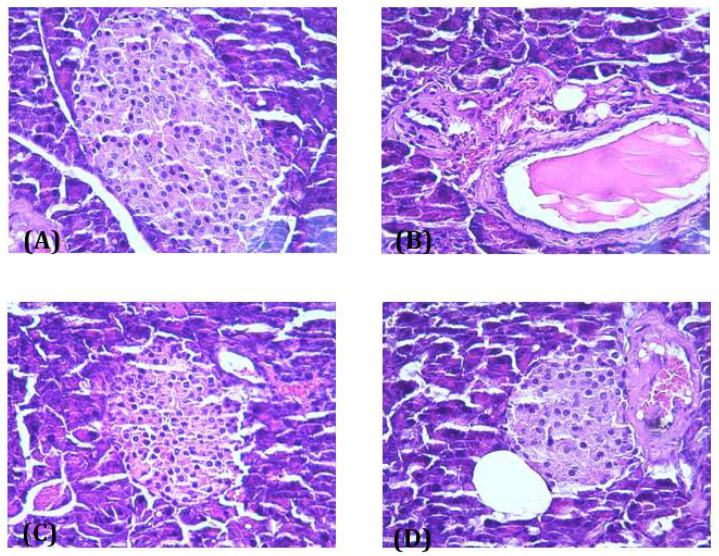
Histopathological observations of pancreatic islets of Langerhans of normal control (**A**), Allx group (100 mg/kg) (**B**), alloxan and EACe (60 mg/kg) (**C**), and Allx and Asc acid (40 mg/kg) (**D**) (400× magnification).

Light microscopic observation of pancreas sections of the normal control group showed normal architecture and appearance of islets of Langerhans, normal acini and islet cells with no structural changes (Figure 10A). However, the islet cells of the Allx group showed pathological changes inboth the exocrine and endocrine parts of the pancreas, represented by vacuolation and a marked decrease inβcells (Figure 10B). Administration of EACe showed a positive appearance of beta cells, and the pancreas appeared similar to the control. Further, most of the islets of Langerhans were intact with no alteration and necrotic changes of the islets of Langerhans (Figure 10C). Likewise, ascorbic acid prevented the severity of degenerative changes of the islets of Langerhans (Figure 10D). 

## 4. Discussion

Herbal medicines have gained popularity as potential therapeutic agents for the prevention and treatment of diverse diseases. It is estimated that about 80% of the world′s population uses natural therapy for medication purposes [43]. Additionally, herbal medicine represents more than 90% of the traditional medicine remedies [29,44]. Medicinal plants have become an immense source for future drug development and human healthcare [45]. Among many herbal medicines, we studied *C. europaea* of Morocco to investigate its preventive effect against diabetes.

Phenolic compounds, as well as flavonoids, are classified as secondary metabolites that have shown therapeutic health benefits and are characterized by their potential effects as natural antioxidants [46]. Phenolic and flavonoid contents of plants may vary due to environmental factors such as sun exposure, soil type, rainfall, and other factors [47]. The findings obtained in the present study showed that the ethyl acetate fraction of *C. europaea* contained the lowest quantity in polyphenols, flavonoids, and tannins. There is a high level of polyphenoland flavonoid contents in *C. europaea*, whichwas reported in an earlier study performed by Amrati et al. [48], who reported 51.42  ±  0.003 mg GA/g of extract of phenolic content and 20.06  ±  0.007 mg QE/g of extract of flavonoid content. This difference could be due to the collection zone, climate conditions, and harvest period [29]. 

Oxidative stress plays a key role in the development of diabetic pathologies [49]. The antioxidant activity determined by the DPPH assay showed that the ethyl acetate fraction (EACe) possessed high antiradical properties in a dose-dependent manner. In this work, EACe has a powerful DPPH free-radical scavenging ability which could be attributable to the presence of phenolic compounds. In the β-carotene bleaching test, the ethyl acetate fraction had a higher antioxidant potential. These findings suggested that *C. europaea* possessed the ability to protect β-carotene against oxidation. Furthermore, the ethyl acetate fraction exhibited the highest FRAPactivity. These findings are in line with previous studies reporting that *C. europaea* has a high antioxidant activity [32,48]. The presence of bioactive compounds such as polyphenols, flavonoids, and tannins, even in lowamounts, may contribute to the antioxidant activity of *C. europaea*.

The glucose diffusion inhibition test was undertaken to perform the effect of aqueous extract and ethyl acetate fraction of *C. europaea* concerning its glucose retardation activity across the dialysis tube. The aqueous extract was not a potent inhibitor of glucose diffusion, while the ethyl acetate fraction of *C. europaea* demonstrated a significant inhibitory effect on glucose movement into an external solution across the dialysis membrane at 60, 120, and 180 min compared to the control. The inhibition of glucose diffusion might be attributed to the physical obstacle presented by fiber particles towards the glucose molecules and the entrapment of glucose within the network formed by fibers [50,51]. Thus, the mechanism of action that could be partly responsible for the postprandial effect is the sequestration of carbohydrates ingested with the meal, thereby retarding their access to digestive enzymes [51]. Previous studies have demonstrated that *C. europaea* showed an inhibitory effect on the intestinal absorption of glucose in vivo [30]. In addition, the retardation of glucose diffusion is also due to the inhibition of α-amylase. In this context, our results revealed that the aqueous extract and ethyl acetate fraction of *C. europaea* inhibited the starch degradation by pancreatic α-amylase in vitro and in vivo. The inhibition of α-amylase activity by *C. europaea* can be attributed to many factors such as fiber concentration and the presence of inhibitors on fibers, which reduce the accessibility of starch to the enzyme, leading to decreased amylase activity [51]. 

The high level of blood glucose generates oxygenfree radicals through glucose autooxidation, which are associated with the pathogenesis of diabetic complications. The present work is the first to report the protective effect of *C. europaea* against alloxan-induced diabetes in mice. The obtained results demonstrated that the ethyl acetate fraction of *C. europaea* prevented alloxan-induced hyperglycemia in mice in terms of antioxidant defense. The pretreatment with 60 mg/kg of EACe prevented the steep onset of hyperglycemia after alloxan injection and maintained blood glucose values slightly close to the normal levels. This effect could be explained by the inactivation of circulating free radicals that quench NO before it reaches βcells, which were producing necrosis [52]. Moreover, NO reacts with superoxide radicals to form the noxious peroxynitrite, a contributing factor to the pathogenesis of diabetes and its complications [53]. Additionally, bodyweight is an important factor that reflects the state of health of experimental mice and the decrease in body weight correlates with defects in body metabolism [54,55]. The reduction inbody weight after alloxan injection was in agreement with previous studies, which attributed this reduction to the elevation of hyperglycemia [56,57]. Interestingly, EACe administration caused a significant increase in body weight. Thus, these positive effects of the ethyl acetate fraction of *C. europaea* were confirmed by histological findings. The protective effect of EACe against the diabetogenic effect of alloxan can be attributed to the presence of antioxidant compounds, which protect βcells against alloxan-induced free-radical production. This hypothesis is supported by the antioxidant activity of EACe confirmed in this paper. 

## 5. Conclusions

The current study demonstrates that *C. europaea* exhibited promising antioxidant and antihyperglycemic activities through inhibiting carbohydrate-digesting enzymes and glucose absorption, displayingits potential preventive role against alloxan-induced diabetes mellitus in mice. These multiple pharmacological profiles may be attributed to the synergistic effect of its natural bioactive compounds. Further, studies could be undertaken to uncover the mechanisms of action involved in the exhibitedantihyperglycemic activity of *C. europaea*. 

## Figures and Tables

**Table 1:** Total phenolic, flavonoid and tannin contents of *C. europaea* (*n* = 3).

Extract	Species	Total Polyphenols (mg GAE/g Extract)	Total Flavonoids (mg QE/g Extract)	Total Tannins (mg GAE/g Extract)
Ethyl acetate fraction (EACe)	*C. europaea*	7.159 ± 0.35	1.523 ± 0.01	0.097 ± 0.002

## Data Availability

All the data supporting the findings of this study are included in this article.
